# Specifics of Cryopreservation of Hydrogel Biopolymer Scaffolds with Encapsulated Mesenchymal Stem Cells

**DOI:** 10.3390/polym16020247

**Published:** 2024-01-15

**Authors:** Marfa N. Egorikhina, Yulia P. Rubtsova, Daria D. Linkova, Irina N. Charykova, Ekaterina A. Farafontova, Diana Ya. Aleinik

**Affiliations:** Federal State Budgetary Educational Institution of Higher Education, Privolzhsky Research Medical University of the Ministry of Health of the Russian Federation (FSBEI HE PRMU MOH), 603600 Nizhny Novgorod, Russia; egorihina.marfa@yandex.ru (M.N.E.); rubincherry@yandex.ru (Y.P.R.); irina-ch0709@yandex.ru (I.N.C.); daleynik@yandex.ru (D.Y.A.)

**Keywords:** cryopreservation, hydrogel scaffolds, stem cells, biopolymers, tissue-engineered constructs

## Abstract

The demand for regenerative medicine products is growing rapidly in clinical practice. Unfortunately, their use has certain limitations. One of these, which significantly constrains the widespread distribution and commercialization of such materials, is their short life span. For products containing suspensions of cells, this issue can be solved by using cryopreservation. However, this approach is rarely used for multicomponent tissue-engineered products due to the complexity of selecting appropriate cryopreservation protocols and the lack of established criteria for assessing the quality of such products once defrosted. Our research is aimed at developing a cryopreservation protocol for an original hydrogel scaffold with encapsulated MSCs and developing a set of criteria for assessing the quality of their functional activity in vitro. The scaffolds were frozen using two alternative types of cryocontainers and stored at either −40 °C or −80 °C. After cryopreservation, the external state of the scaffolds was evaluated in addition to recording the cell viability, visible changes during subsequent cultivation, and any alterations in proliferative and secretory activity. These observations were compared to those of scaffolds cultivated without cryopreservation. It was shown that cryopreservation at −80 °C in an appropriate type of cryocontainer was optimal for the hydrogels/adipose-derived stem cells (ASCs) tested if it provided a smooth temperature decrease during freezing over a period of at least three hours until the target values of the cryopreservation temperature regimen were reached. It was shown that evaluating a set of indicators, including the viability, the morphology, and the proliferative and secretory activity of the cells, enables the characterization of the quality of a tissue-engineered construct after its withdrawal from cryopreservation, as well as indicating the effectiveness of the cryopreservation protocol.

## 1. Introduction

The dynamic development of regenerative medicine is currently inextricably linked with increased tissue engineering success resulting from the use of complex tissue-engineered products with three-dimensional structures rather than through the application of cultures of individual cells.

Tissue engineering is defined as the use of cells, biomaterials, and suitable molecular or physical factors, individually or in combination, to repair or replace tissues to improve clinical outcomes [1]. The successful development of tissue-engineered constructs (TECs) depends on two key factors:

(1) The use of appropriate cells capable of regenerating or replacing the tissue for the restoration the product is intended for [2,3];
(2) The correct microenvironment acting as an artificial cellular niche that can be constructed by, for example, scaffolds or the use of bioink for bioprinting. It should be noted that the “correct microenvironment” corresponding to the niche concept involves not just one that allows for the retention of cell viability, but also enables the initiation of various cellular events depending on signals coming from the scaffold, both mechanical and biochemical [4,5].

Thus, the creation of a tissue-engineered construct capable of providing fully fledged tissue restoration is a time-consuming and expensive process requiring the coordinated activities of specialists in many fields. As a rule, the successful development of new types takes several years. At the same time, it is known that the resulting tissue-engineered products have a short lifespan. This is the result of the TEC being a living product. Cellular events continue within it, such as the build-up of cell mass, the secretion of growth factors by the cells, and the interaction of cells among themselves, involving the regulation of cellular processes [6,7]. Furthermore, the artificial extracellular matrix of the TEC can also affect cellular processes, e.g., it may influence migration and proliferative activity, phenotype change, the cells’ choice of a resting or self-renewal state, or their direction of differentiation [8,9]. At the same time, processes similar to natural “dynamic reciprocity” can occur in a tissue-engineered construct; the cells remodel their microenvironment, i.e., the scaffold, changing its structure and properties [10,11]. All these processes cannot continue indefinitely, as they take place only under certain conditions. Therefore, the lifespan and the timeframe over which the TEC is suitable for insertion into the recipient are quite short and often amount to only a few days. By contrast, the manufacturing time of the construct itself is long and varies from a few days to several weeks, sometimes, even months, depending on the technology.

By contrast, when vital organs are damaged, e.g., when extensive skin burns have occurred, minimizing the time elapsed between the traumatic event and treatment (transplantation) is crucial for the patient’s life. Often this period is measured in hours or, at a maximum, in days, and there is only an extremely short period available for decision-making. This is when the possibility of immediate transplantation of cellular products and tissue-engineered constructs for patients comes to the fore. The availability of tissue-engineered products at the required time can be ensured through effective cryopreservation. Such an ability to retain the integrity of tissue-engineered products over a long period of time will allow the creation of biobanks, ensuring the safe storage of tissue-engineered structures that can be used immediately in the case of clinical need [12].

In this regard, the development of optimized protocols for the cryopreservation of tissue-engineered constructs is a necessary prerequisite for the commercialization and further development of regenerative medicine. Despite the cryopreservation of complete TEC structures being more complicated than the cryopreservation of individual cells or cell suspensions, recent studies have proved the fundamental possibility of such cryopreservation and the possibility of successful use of the products after the defrosting procedure [13,14].

Interestingly, back in 2003 it was shown that SaOS-2 cells immobilized on two-dimensional or three-dimensional carriers based on oly (lactide-co-glycolide) exhibited greater viability after cryopreservation with 10% DMSO than a simple suspension of these cells under the same conditions [15]. However, despite early successes, the issue of the cryopreservation of TECs has proven to be much more difficult to solve. When such cells are encapsulated within three-dimensional structures, a significant problem arises as a result of uneven penetration of the cryoprotectant. Thus, a sufficient amount of cryoprotectant may not reach cells located in the depths of the TEC, while at the same time, cells located in the superficial layers of the construct may be subject to cytotoxic effects (due to exposure to high concentrations of the cryoprotector). Another problem for 3D structures is the uneven rates of cooling and heating of different regions. This can result in uneven expansion or contraction of the scaffold, having direct negative effects on the cells (including on their adhesion) [13]. Any extracellular matrix (ECM) produced by the cells during the cultivation of the TECs prior to cryopreservation can also play an important role in the success of cryopreservation. Thus, while in one situation, the accumulation of ECM can play a positive role, providing additional protection for the cells [16], a different situation such a buildup can interfere with the permeability of the structure to the cryoprotectant, preventing it from reaching the cells and, as a result, negatively affecting their viability after cryopreservation [15]. Therefore, when developing a protocol for the steps preceding TEC cryopreservation, it is important to take into account the type of cells and their activities in relation to the accumulation of any ECM, as well as the composition and density of the accumulated ECM.

Of course, the carrier itself also plays an important role in the success of TEC cryopreservation. Its composition and structure can influence the safety of the TEC cellular component. Indeed, in a work by A.I. Pravdyuk [17], it was shown that alginate hydrogel, itself, has cryoprotective properties. Similar cryoprotective properties have been demonstrated for hydrogels based on trehalose; D. Diaz-Dussan et al. [18] showed that trehalose-based scaffolds can act as cryoprotective agents by being able to mitigate physical damage to the cells during freezing and thawing through their influence on the formation and growth of ice crystals. In a work by P. F. Costa et al. [19], it was shown that the structure of a starch–polycaprolactone scaffold determined the content of the cellular material retained by the TECs after cryopreservation and thus the viability of the MSCs; in porous scaffolds, both the amount of DNA and the viability of the MSCs were almost three times higher than in non-porous disks of the same composition. The influence of the internal architecture of scaffolds on the success of cryopreservation was demonstrated in the work of O. Batnyam et al. [20]. Here, in polyurethane scaffolds consisting of randomly oriented nanofibers, the cell viability after cryopreservation was significantly higher than that in scaffolds with highly ordered nanofibers.

A rather difficult issue in TEC cryopreservation is the standardization of cryopreservation protocols. As a rule, typical TEC cryopreservation protocols are based on cell suspensions and include several stages:

1. Preparatory stage—replacing the growth medium with a cryopreservation medium containing the cryoprotector, followed by incubation at positive temperatures (to allow penetration of the structure with the cryoprotector before freezing);
2. Freezing stage—at this stage, the choice of freezing speed (vitrification or gradual decrease in temperature) is critical;
3. Cryostorage stage—the choice of an optimal temperature regimen for this stage and its stability can determine the maximum duration of successful cryostorage;
4. Thawing stage—it is important to ensure thawing of the sample without allowing recrystallization of the liquid. This stage also includes the subsequent washing of the cryopreservative from the sample;
5. Acclimatization stage—not used in all protocols, this stage includes incubation of the product in order to restore the functional activity of its cellular component [21].

Almost all stages of the different protocols involve significant differences, so cryopreservation protocols require careful preparation, since their effectiveness depends on many parameters: the cells’ cryosensitivity, the scaffold architecture, the cells’ culture conditions before freezing, the different cryoprotectant compositions, etc. [13,22].

The most important aspect of TEC cryopreservation is the evaluation of its effectiveness. In the vast majority of studies, the authors focus on cell viability as the key evaluation criterion [20,23,24,25]. However, there is no consensus on what level of cell viability is sufficient. Thus, restoration of about 50% of the cells is commonly used, as described in a number of works [12,20,26]. Unfortunately, this level is unlikely to be sufficient to achieve a significant clinical effect. This view is supported by the evidence that for human MSC products, the clinical acceptance limit for post-thaw cell viability is at least 70% [27,28]. It is also undeniable that the clinical effectiveness of a TEC is determined not only by its cell viability, but also, importantly, by the cells’ functional activity. Depending on the type of cell, this can include their proliferative and secretory activity, adhesion ability, and differentiation potential [18,29]. It is also important not to forget the need to preserve the properties of the scaffold carrier after cryopreservation. For example, cryopreservation can be accompanied by the formation of microcracks that arise due to the expansion of ice in the pores of the structure [14].

In general, the complexity and diversity of TECs make it impossible to use just a single cryopreservation and evaluation protocol for the different types. In turn, having to develop a separate protocol for each specific product greatly complicates standardization. Therefore, currently, we are essentially still at the stage of accumulating knowledge about the cryopreservation of TECs and developing criteria for evaluating the effectiveness of different techniques. 

We previously developed a hydrogel polymer scaffold capable of acting as an artificial cell niche for adipose-derived stem cells (ASCs). We have also shown the in-principle possibility of its cryopreservation for three months, whilst still retaining the viability and proliferative activity of the ASCs it contains [30,31]. We anticipate that in the future, the TEC we have developed will be appropriate for use with skin equivalents, including those intended for the restoration of wounds in patients with burns. The latter situation requires rapid product availability “as needed”, which can be achieved through effective cryopreservation. The purpose of the current work was to study the effects of TEC cryopreservation on the functionality of ASCs encapsulated within such scaffolds when using various different cryopreservation protocols.

## 2. Materials and Methods

### 2.1. Obtaining the ASC Cell Culture

The study used mesenchymal stem cells of passage 4 isolated from human adipose tissue (ASCs). The starting material for the ASC culture was adipose tissue obtained after cosmetic plastic surgery. Voluntary, informed consent was obtained from each donor. The research protocol was approved by the FSBEI HE PRMU MOH local ethics committee (protocol No. 5 of 10 March 2021; protocol No. 9 of 30 June 2023).

Adipose tissue collected in the operating room was placed in a vial with transport medium and taken to the biotechnology laboratory of the FSBEI HE PRMU MOH University Clinic. Subsequent work was run in vertical laminar flow under sterile conditions. The cells were extracted using enzymatic processing with collagenase type I (Stemcell Technologies, Vancouver, BC, Canada) for an hour at +37 °C and cultivated in complete growth medium at absolute humidity, +37 °C, and 5% CO_2_. The complete growth medium had the following composition: MesenCult™ MSC Basal Medium (Human), MesenCult™ MSC Stimulatory Supplement (Human), glutamine, and penicillin/streptomycin antibiotics. The media and reagents were sourced from Stemcell Technologies (Vancouver, BC, Canada), and the plastic consumables were those supplied by Costar (Washington, DC, USA). After reaching a subconfluent (60–70%) monolayer, the cultures were re-plated (at a density of 5 × 10^3^/cm^2^). Cultures from the third such passage were used for the experiments. The resulting cell suspension was seeded into culture vials (Corning, NY, USA) for further cultivation. The cells were cultured in CO_2_ incubator conditions (5% CO_2_, +37 °C, absolute humidity), changing the medium twice a week. Before use in the experiment, the cell cultures were tested for sterility and contamination with mycoplasmas and viruses.

Thus, the cells used for the formation of the scaffolds met the criteria specified for mesenchymal cells by the International Society for Cellular Therapy [32]. These cells expressed CD 105+, CD 90+, CD 44+, and CD 73+ and did not express CD 14-, CD 45-, or HLA DR-. For the experiments, we used the monoclonal antibodies CD 105 PE (cat: B76299), CD 90 FITC (cat: IM1839U), CD 44 FITC (cat: IM1219U), CD 73 PE (cat: 550257), CD 14 PC5 (cat: A07765), CD 45 PC5 (cat: A07785), and HLA-DR PC7 (cat: A40579) (all from Becton Dickinson, Franklin Lakes, NJ, USA) with the corresponding isotypic controls (cat: A07795, A09142, A07798, 737662), in a BD FACS CANTO II flow cytometer system (Becton Dickinson, Franklin Lakes, NJ, USA), with the monoclonal antibodies being used at a concentration of 1%. The cells used also spread well on plastic surfaces and were capable of differentiating in any of three directions, being adipogenic, osteogenic, and chondrogenic, as demonstrated using the Hyman Mesenchymal Stem Cell Functional Identification Kit (R and D systems, Minneapolis, MN, USA). Prior to introduction into the scaffold composition, the cells had a viability of 98–99%.

### 2.2. Formation of Scaffolds with Encapsulated ASCs

To form a scaffold with encapsulated ASCs, we used the technique we have described previously [33]. The composite used to form the hydrogel scaffold contained PEGylated human plasma, cryoprecipitate proteins, and type I collagen. To encapsulate the cells within the scaffold, a suspension of the ASCs in phosphate buffer (PBS) was introduced into the composite. The cell concentration was 1.2 × 10^5^/1 mL of the composite. The scaffolds were formed under conditions of enzymatic hydrolysis through the addition of a thrombin–calcium mixture to the composite. The resulting scaffolds were cultured, immersed in α-MEM complete growth medium in a CO_2_ incubator (conditions: +37 °C; 5% CO_2_ content; absolute humidity).

### 2.3. Cryopreservation and Scaffold Recovery Procedures

On the third day of cultivation, the cell scaffolds were cut into equal-sized fragments using a circular template with a diameter of 1 cm. Some of the fragments continued to be cultured for another 72 h (up to the sixth day), while the others were cryopreserved. For this, the scaffold fragments were placed in cryoprobes with a cryoprotective medium containing 10% DMSO (Sigma-Aldrich, Darmstadt, Germany). To ensure slow cooling, the cryoprobes were placed into cryocontainers of types typically used for the freezing and cryopreservation of cell cultures. Two types of cryocontainers were used. Cryocontainers of type No. 1 were made of polyethylene foam with a thermally conductive semiconductor core to stabilize the cooling temperature. Cryocontainers of type No. 2 were plastic foam containers with no core. The cryocontainers of each type containing scaffold samples were placed into freezers, either at −80 °C or at −40 °C (Nuve DF 290, Nuve FR 290 Sanayi Malzemeleri ve Ticaret A.S., Turkey). After 1 month, the scaffolds were removed from cryopreservation and returned to a temperature of +37 °C. The thawed samples were washed with phosphate buffer, covered with complete growth medium, and cultured under standard CO_2_ incubator conditions.

### 2.4. Comparative Characteristics of the Scaffold Cellular Component before and after Cryopreservation

ASCs located in the scaffold structure were compared on the 3rd (72 h) and 6th (144 h) days of cultivation without cryopreservation and after 24 and 96 h of cultivation following cryopreservation under different temperature regimens.

#### 2.4.1. Microscopic Assessment of Cell State in Scaffold Structure

To visualize the cells using light microscopy, a Leica DMIL LED (Leica Microsystems, Wetzlar, Germany) microscope equipped with a video camera was used along with Leica LAS V4.13 image visualization software.

#### 2.4.2. Evaluation of the Number and Viability of Cells in the Scaffold Structure

The state of cells carrying out vital activity within the scaffold structure was evaluated using fluorescence microscopy on a multifunctional imager, the Cytation 5 with Gen 5 Image+ software; (BioTek, Winooski, VE, USA) using the fluorochromes Hoechst 33342 (BD, Franklin Lakes, NJ, USA) and TO-PRO3 Ready Flow Reagent (Invitrogen by Thermo Fisher Scientific, Waltham, MA, USA).

This method is based on the use of intravital staining of nuclei with the Hoechst 33342 fluorochrome that is highly specific for double-stranded DNA molecules (377 nm excitation wavelength and 447 nm emission wavelength), followed by counting the cells in 10 visual fields and using the Z-stack function. The total number of cells was estimated per 1 mm^3^ of scaffold.

To mark and estimate the number of dead cells in the scaffold structure during cultivation and after cryopreservation, the fluorochrome TO-PRO™3 Ready Flow™ Reagent Invitrogen™ was used as it stains the nuclei of dead cells (586 nm excitation wavelength, 647 nm emission wavelength). The difference results from this fluorochrome being unable to penetrate the cytoplasm of viable cells, although it too has specificity for double-stranded DNA molecules.

For staining, the scaffold fragment was placed in the well of a 24-well tablet, and 2 mL of culture medium and 1 mL of Hoechst 33342 solution at a concentration of 10 µg/mL were added. The sample was placed in a thermostat and incubated for 15 min at 37 °C. Then, one drop of fluorochrome TO-PRO™3 Ready Flow™ was added to the well and incubated at room temperature in the dark for 15 min. After incubation, the scaffold fragment was washed twice with a phosphate buffer.

The combination of the two fluorochromes, Hoechst 33342 and TO-PRO™3 Ready Flow™ Reagent Invitrogen, allows for estimation of the ratio of living and dead cells. The number of dead cells was expressed as a percentage of the total number of cells.

#### 2.4.3. Assessment of Secretory Activity of ASCs in Scaffolds

The secretory activity of the ASCs in each scaffold sample was assessed by means of the levels of VEGF and IL-8 accumulated in the conditioned medium during scaffold cultivation. Fragments of scaffolds before and after cryopreservation were cultured in the wells of a culture tablet, each fragment in 2 mL of medium. Sampling was carried out within the control periods: 3 days after separation into fragments, 24 h after defrosting, 96 h after defrosting. The selected aliquots were frozen and stored at −40 °C for no more than 2 months. The amounts of VEGF and IL-8 in the medium were measured using enzyme immunoassay (EIA). The analysis was carried out in accordance with the manufacturer’s protocol. The amount of detectable protein of each type was measured in pg/mL in accordance with calibration curves constructed from the optical densities of VEGF standards using the VEGF-ELISA-BEST kit (cat. DVE00, R&D Systems, Bio-Techne^®^, Minneapolis, Minnesota, USA) and the IL-8 standards from the Human IL-8 ELISA Kit (cat. BMS204-3 Thermo Fisher Scientific, Waltham, MA, USA). When setting up the EIA, the optical density was measured using an INFINITE F50 microplate reader (Tecan Austria GmbH, Grödig, Austria) at 450 nm. Measurements were run according to the manufacturer’s protocol.

### 2.5. Estimation of Cooling Rate in Cryocontainers during Freezing

The cooling rate in cryocontainer types No. 1 and No. 2 during freezing was assessed using a LogTag^®^ TREL30-16 (LogTag Recorders Limited, Rosedale, Auckland, New Zealand) temperature recorder with a remote sensor. To adjust the temperature recorder, LogTag Analyzer 3 software was used. The following parameters were configured: measurement start on pressing the “Start” button; measurement stop by pressing the “Stop” button; 5 min recording intervals with no limits set for overall recording duration; °C temperature units. After setting up, the temperature recorder sensor was placed into the relevant cryocontainer (No. 1 or No. 2). Then, after pressing the “Start” button, the cryocontainer movements were reproduced as the scaffold samples were frozen. After reaching the appropriate cryopreservation temperature, the cryocontainer with the thermal sensor was removed from the freezer and measurement recording was stopped. Measurements of data from each cryocontainer type were repeated four times for each temperature regimen (−40 °C and −80 °C).

### 2.6. Statistical Analysis

Statistical analysis was performed using the STATISTICA 6.0 (Dell Technologies Inc., Round Rock, TX, USA) software package. The research results were processed with nonparametric statistical methods, using the Wilcoxon paired comparison test. The results are presented as the mean (M) ± m. The level of significance was set as follows: *p* < 0.05; *p* < 0.01; *p* < 0.001. Statistical significance of the results was judged at *p* < 0.05. The result was judged as a higher or extremely significant difference at *p* < 0.01 and *p* < 0.001.

## 3. Results and Discussion

**Figure 1:**
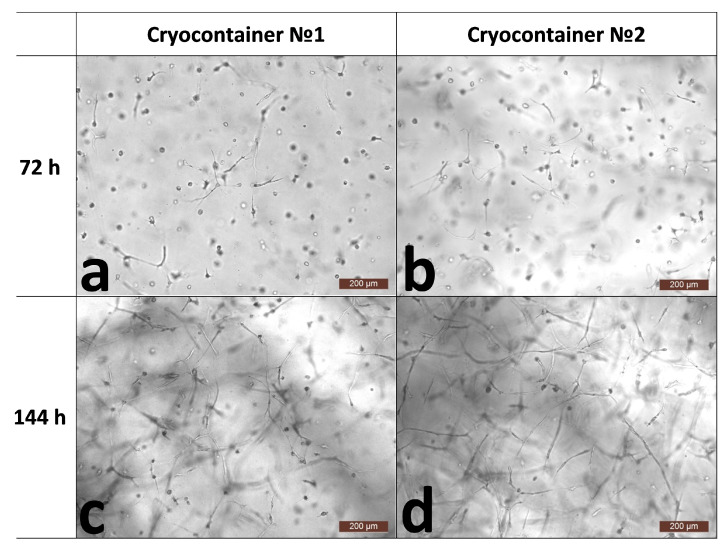
Representative photographs of the state of the cells within the scaffold structure without freezing. (**a**,**b**)—72 h of cultivation; (**c**,**d**)—144 h of cultivation.

The hydrogel biopolymer scaffolds with encapsulated ASCs were cultured for 6 days (144 h) under standard conditions (+37 °C; 5% CO_2_ content; absolute humidity). The proliferative activity and viability of the cells in the scaffolds without cryopreservation were evaluated. It was shown that when cultured for 3 (72 h) to 6 days (144 h), the total number of cells increased by more than 1.5 times. The percentage of dead cells before cryopreservation did not exceed 1%, and in general, their number could be characterized as “single or none in the field of vision” (Table 1). This indicated that the cells in the scaffolds before cryopreservation had evident proliferative activity and high viability. Before cryopreservation (by 72 h), the cells had formed outgrowths and intercellular contacts inside the scaffold (Figure 1a,b). When cultured for up to 144 h without cryopreservation, the cells also showed active three-dimensional growth, with the previously formed outgrowths significantly lengthening as the cells formed a cellular network (Figure 1c,d). It was noted that by the sixth day of cultivation, the cellular scaffold samples had become smaller (Figure 4A). Also, the scaffold samples became less transparent and acquired a “whitish” color. We have previously shown that the cells actively growing within a scaffold affect the scaffold itself. They are able to transform their microenvironment, e.g., by secreting various proteins. It was shown that during ASC cultivation, the scaffold structure became compacted [30], presumably causing its external changes.

One month after cryopreservation, the scaffolds were thawed. It is important to note that according to the literature data, a short acclimatization period after cell defrosting contributes to the restoration of the functional potential of the MSCs [34,35,36]. Most often, a 24 h period is specified for such thawed material reactivation [37]. In our study, we therefore also ran a restoration or acclimatization procedure for the thawed scaffolds with encapsulated ASCs, cultivating them under standard CO_2_ incubator conditions for 24 h before the analysis started.

Analysis of the total number of cells per 1 mm^3^ of scaffold after cryopreservation and acclimatization (72 h) showed that the number of cells per unit volume had increased compared to their number before cryopreservation. These changes were observed for samples frozen both at −40 °C and at −80 °C (Table 1). In particular, the number of cells in the scaffolds frozen and stored in containers of type No. 1 had increased by 30%, while those in containers of type No. 2 had increased by more than 50%. This cell number increase definitely could not be associated with their proliferative activity because during cryopreservation, they had been in a state of stasis, and the subsequent acclimatization period (24 h) was too short for the cells to be able to increase their number so significantly due to division. It should also be kept in mind that the complete functional activity of the cells is not restored immediately after cryopreservation, but only after a certain period of adaptation. Thus, the cell number per unit volume of scaffold had increased, not due to their proliferative activity, but as a result of scaffold “shrinkage”. We had previously observed the phenomenon of the osmotic shrinkage of hydrogel scaffolds under longer cryopreservation periods from 3 to 12 months. In our publication, the causes and mechanisms of this phenomenon were analyzed in detail [31]. Thus, the cryoprotectant, not being limited by any membranes, replaces a large volume of the liquid in the hydrogel, leading to the “shrinkage” of the hydrogel structure. The macroscopic state of the scaffolds confirmed this “shrinkage” effect. Therefore, when the condition of the samples was assessed visually, it was noted that before freezing (72 h), scaffold fragments created according to the template had 11 mm diameters (Figure 4A), but this diameter decreased to approximately 6–8 mm after freezing and defrosting (Figure 4B). Thus, the scaffold size changes show that the structure’s density also changed. As the number of cells is calculated per scaffold volume unit (in mm^3^), this explains the increase in cell numbers per unit volume after cryopreservation. It should also be noted that shrinkage of the scaffolds stored in containers of type No. 2 was presumably larger than of those in containers of type No. 1, since the increase in cells numbers in them was more than 20% greater. However, it should be noted that during the visual external evaluation, no differences were evident between samples stored in cryocontainers of types No. 1 and No. 2.

**Figure 2:**
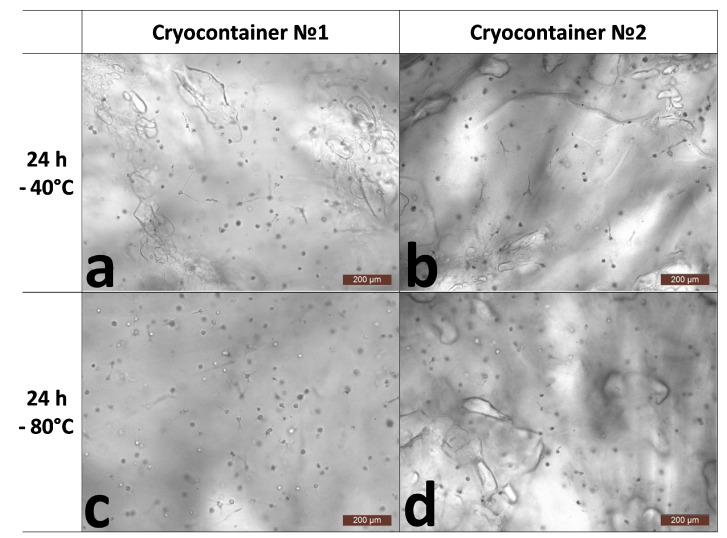
Representative photographs of the state of cells inside the skeleton structure 24 h after thawing. (**a**)-after cryopreservation in cryocontainer No. 1 at −40 °C; (**b**)-after cryopreservation in cryocontainer No. 2 at −40 °C; (**c**)-after cryopreservation in cryocontainer No. 1 at −80 °C; (**d**)-after cryopreservation in cryocontainer No. 2 at −80 °C.

Despite the physical changes in the scaffolds, cell viability remained at a very high level and was at least 95% (Table 1). During microscopic observation, it was noted that after the acclimatization period (24 h), the overall state of the cells was comparable for all the scaffold samples (Figure 2). Both cells with single or several outgrowths and cell spheroids were observed. However, during the microscopy, we noticed that the condition of the scaffolds themselves was somewhat different. Scaffolds stored at −40 °C (containers No. 1 and No. 2) and at −80 °C (container No. 2) had defects on the surface that can be described as oval “shells” of various sizes (Figure 2a,b,d). The described defects were essentially almost absent from samples stored at −80 °C in containers of type No. 1 (Figure 2c).

**Figure 3:**
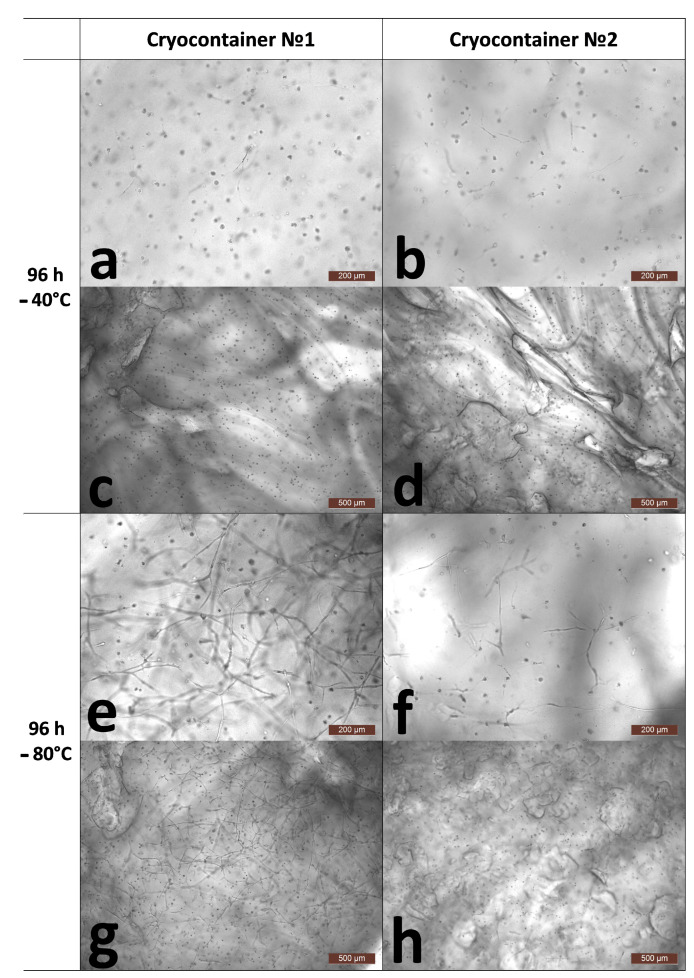
Representative photographs of the state of cells inside the skeleton structure 96 h after thawing. (**a**,**c**)-after cryopreservation in cryocontainer No. 1 at −40 °C; (**b**,**d**)-after cryopreservation in cryocontainer No. 2 at −40 °C; (**e**,**g**)-after cryopreservation in cryocontainer No. 1 at −80 °C; (**f**,**h**)-after cryopreservation in cryocontainer No. 2 at −80 °C; (**a**,**b**,**e**,**f**)-scaffold surface; (**c**,**d**,**g**,**h**)-scaffold interior.

**Figure 4:**
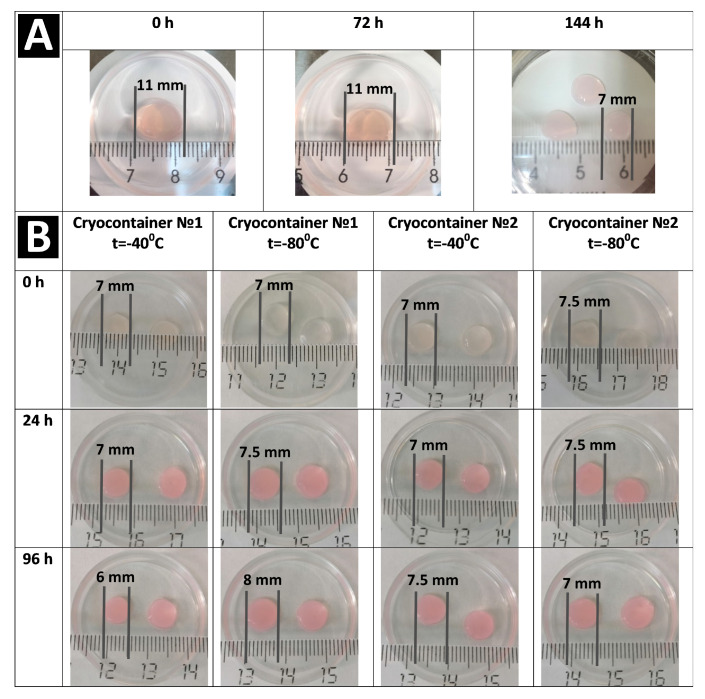
Changes of the appearance and size of the scaffold. (**A**) no cryopreservation. (**B**) after cryopreservation.

Further cultivation of the scaffolds for 96 h after thawing showed different results in terms of cell numbers, viability, and their morphology. Thus, analysis of the number of cells per 1 mm^3^ of scaffold samples stored at −40 °C showed a decrease after 96 h compared to the results of the analysis run 24 h after defrosting (Table 1). The cells’ viability had also changed. The cell viability for scaffolds stored in cryocontainers of type No. 1, although tending to decrease, remained at a fairly high level of about 94%. The viability of cells stored in cryocontainers of type No. 2 was significantly lower, no more than 88%. Upon the microscopic assessment of the state of the cells, similar patterns were observed for the stored scaffolds regardless of their cryocontainer type. A proportion of the cells had formed long outgrowths and intercellular contacts (Figure 3e–g). At the same time, quite a lot of cells had become “cell spheroids”, evidencing that they had either died or were on the verge of death (Figure 3a–d,h). It is known that after cryopreservation, a significant loss of MSCs (up to 20%) occurs against the background of the restoration of viable cells. Apoptotic and necrotic pathways are activated in cells 6 to 48 h after thawing in response to their previous exposure to low temperatures [38], and cryopreserved MSCs have a higher percentage of apoptotic cells than MSCs from fresh live cultures [39]. Considering the condition of the cells, their decreased viability, their range of morphology, and zero increase in numbers, it can be argued that the cells retained no evident proliferative activity. The decrease in the number of cells per unit of scaffold volume after 96 h of cultivation can be explained by the fact that over the time of cultivation, the scaffold, which is a hydrogel, regained its volume of liquid and “thus expanded” after its previous “shrinkage”. This was confirmed by microscopic observation of the scaffold surfaces. Figure 3c,d show a significant increase, i.e., stretching, of the scaffold structure defects described earlier (24 h after defrosting). Thus, against the background of inhibition of the cells’ proliferative activity, “expansion” of the scaffold structure led to the apparent decrease in total cell numbers as calculated per unit volume. However, it should be noted that the increase in volume of the scaffolds was not so significant as to be noticeable visually upon external evaluation (Figure 4).

A completely different picture was observed when assessing the number and condition of cells after cryopreservation of scaffolds frozen and stored at −80 °C. Thus, in scaffolds stored in container type No. 1, the number of cells during cultivation for 1 (24 h) to 4 (96 h) days after defrosting increased by almost 38% against the background of scaffold “expansion” (Table 1 and Figure 3e,g). This testified that these cells had retained a high proliferative ability. Microscopy showed that they had multiple branching outgrowths and had formed cellular networks comparable to the cellular network formed by cells in a scaffold not subjected to cryopreservation (144 h). It should be noted that a small number of “cell spheroids” was also observed in the microscope field of view. However, cell viability remained at the level observed after acclimatization (24 h), being greater than 96% (Table 1).

The condition of the scaffolds and cells after cryopreservation in container type No. 2 at −80 °C and after subsequent cultivation differed from that of the scaffolds stored using container type No. 1 under the same conditions. Thus, the total cell count in the scaffold samples during the cultivation period after withdrawal from cryopreservation and acclimatization did not change (Table 1). However, the cell viability decreased significantly and became less than 90% (96 h). The microscopic picture of the state of the cells matched the results of the analysis. Most of the cells demonstrated recovery, but to varying degrees. Thus, a number of the cells had multiple and branched outgrowths and had formed intercellular contacts. However, other cells were only just beginning to spread out to form outgrowths. Against the background of the recovering cells, cell spheroids were also observed. Thus, considering the quantitative analysis data and the microscopic appearance of the cells, it can be said that most of the cells had retained their proliferative activity. However, the degree of proliferative activity was reduced compared to that of the samples subjected to cryopreservation in cryocontainer type No. 1. It was, therefore, sufficient to compensate for the scaffold “expansion” but insufficient to cause an increase in cell numbers per unit volume.

The macroscopic picture of the scaffold condition used at all stages of the study after cryopreservation, as assessed visually, was comparable for both types of cryocontainers and both temperature regimens (Figure 4B).

It is known that the regenerative effect in MSCs mainly depends on the cells’ paracrine functions [40,41]. There is no doubt that one of the key factors for the successful application of tissue-engineered constructs will also be fulfilment of the paracrine functions of the cells. In this regard, when assessing TEC quality after cryopreservation, it is important to characterize the secretory activity of the cells. It is known that MSCs secrete a fairly wide range of growth factors, cytokines, and extracellular matrix proteins [42,43,44,45,46,47]. One of the key growth factors secreted by stem cells is VEGF (vascular endothelial growth factor). This growth factor is a mitogen, specific for endothelial cells; an inducer of angiogenicity; and a mediator of vascular permeability [48]. VEGF is directly involved in the process of wound healing and tissue repair [49,50,51].

**Figure 5:**
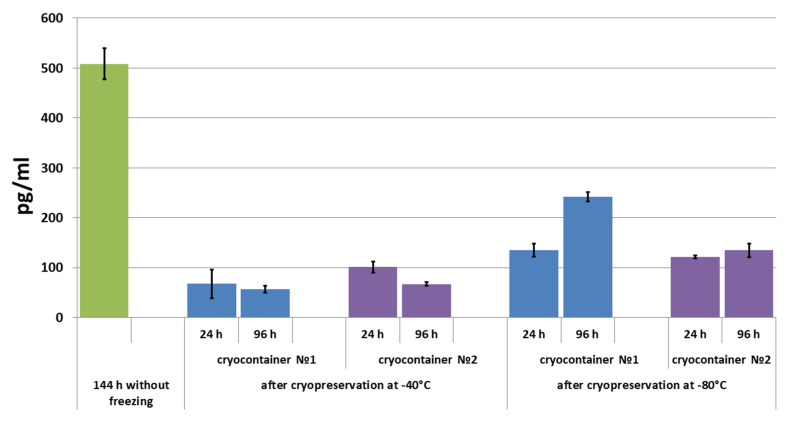
VEGF concentration in conditioned medium during scaffold cultivation before and after cryopreservation.

It was shown that during scaffold cultivation for 3 to 6 days without cryopreservation, the cells actively secreted VEGF, and it accumulated in the conditioned medium (Figure 5, Graph 144 h).

After 24 h of scaffold cultivation following cryopreservation, VEGF levels were evaluated in the conditioned medium of all the studied samples. The highest level of VEGF was observed in the conditioned medium of samples stored in container type No. 1 at −80 °C. The VEGF concentration in the conditioned medium of samples stored at −80 °C in cryocontainer type No. 2 was almost at the same level. In the conditioned medium of samples stored at −40 °C, the evaluated concentration of VEGF was less than that in the conditioned medium of scaffolds stored at −80 °C. When scaffold cultivation was continued for up to 96 h, it was shown that the level of the growth factor that could be determined decreased in the conditioned medium of scaffolds previously stored at −40 °C. A different picture was observed when the VEGF concentration was evaluated in the conditioned medium of samples stored at −80 °C (Figure 5). However, in the medium from scaffolds stored in cryocontainer type No. 2, the VEGF concentration between one (24 h) and four (96 h) days of cultivation after cryopreservation increased slightly (by less than 12%). By contrast, the VEGF concentration increased by almost 80% in the conditioned medium of samples stored in cryocontainer type No. 1 during the same cultivation period. It should be noted that following cryopreservation, the studied factor level did not reach the level observed in the conditioned media of scaffolds not subjected to the cryopreservation procedure.

A comparable pattern was observed when interleukin-8 (IL-8) was evaluated in the conditioned medium. IL-8 is one of the most important components of the MSC secretome (IL-8). It is a pro-inflammatory cytokine with its main function being to attract neutrophils and macrophages to inflammation foci [52]. In addition, IL-8 is involved in the cellular aging processes [53], and it is able to stimulate VEGF production, therefore exhibiting proangiogenic properties [52,54,55]. Hans-Oliver Rennekampff M.D. et al. showed that IL-8 is involved in all stages of the regenerative process during wound healing [56]. The ability to secrete growth factors, including VEGF and IL-8, is quite often used by researchers to assess the quality of the cellular component of tissue-engineered constructs, including after cryopreservation [22,57].

**Figure 6:**
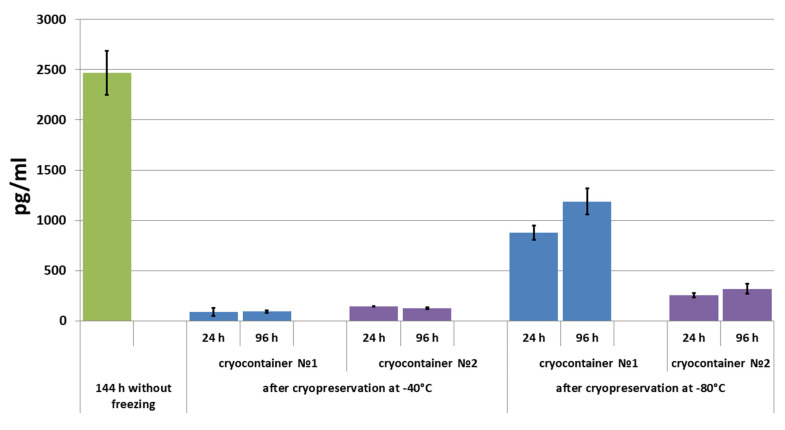
IL-8 concentration in conditioned medium during scaffold cultivation before and after cryopreservation.

It was shown that without prior cryopreservation, during scaffold cultivation under standard conditions (+37 °C; 5% CO_2_ content; absolute humidity) for three days (for 72 h to 144 h), IL-8 accumulated in the conditioned medium (Figure 6). After cryopreservation and 24 h of cultivation in a conditioned medium, the levels of IL-8 from all the studied samples were evaluated. While its level was quite low in the conditioned medium from samples stored at −40 °C, it was significantly higher in the medium of samples stored at −80 °C. Furthermore, the IL-8 concentration in the conditioned medium of samples stored in container type No. 1 (−80 °C) was almost 3.5 times higher than that in the samples stored in container type No. 2 (−80 °C).

With the subsequent cultivation of up to 96 h, almost no further changes were observed in the IL-8 concentration in the conditioned medium of samples stored at −40 °C. It should be noted that in the medium of samples stored in cryocontainer type No. 2, a tendency towards decreasing detectable cytokine levels was observed (Figure 6). Completely different dynamics were observed in the conditioned medium of samples stored at −80 °C. Thus, under these conditions, the IL-8 level in the conditioned medium increased by 35% during the three days of cultivation of samples frozen in cryocontainer type No. 1. In the medium of samples stored in cryocontainer type No. 2, the levels of IL-8 also increased, but only by 25% after three days. In addition, the concentration of IL-8 that accumulated in the medium (by 96 h) of samples stored in cryocontainers of type No. 1 was almost four times higher than the evaluated concentration in the medium of samples stored in cryocontainers of type No. 2 (Figure 6). As with the results obtained when evaluating VEGF levels in the conditioned media, in no case did the IL-8 level in the medium of scaffolds that had been cryopreserved reach the level observed in the conditioned medium of scaffolds not subjected to the cryopreservation procedure.

Summarizing the results obtained, it can be seen that the maximum secretory activity retention of ASCs cultured in scaffolds after cryopreservation was observed in samples stored in cryocontainers of type No. 1 at −80 °C. This is consistent with the data described above, demonstrating that these freezing and cryopreservation conditions ensure better retention of cell viability and proliferative activity. The results of the cryopreservation of scaffolds at −40 °C were unsatisfactory. Under this storage regimen, the growth as well as the proliferative and secretory activity were disrupted after such cryopreservation in both types of cryocontainers. However, it should be noted that the samples stored in cryocontainer type No. 1 showed slightly better results compared to the samples stored in cryocontainer type No. 2 in retaining cell viability. By contrast, slightly better results were obtained for samples stored in cryocontainer type No. 2 at −80 °C; however, they were significantly inferior to the results of storage at the same temperature regimen in cryocontainer type No. 1. It is important to note that in the latter case (cryocontainer type No. 2, −80 °C), cell viability significantly decreased during cultivation after cryopreservation (Table 1). Thus, it has been shown that a −40 °C temperature regimen is not applicable for the cryopreservation of the studied hydrogel biopolymer scaffolds with encapsulated ASCs. By contrast, a temperature regimen of −80 °C allows the cells encapsulated in the scaffold to retain high viability, active growth, and high proliferative and secretory activity.

**Figure 7:**
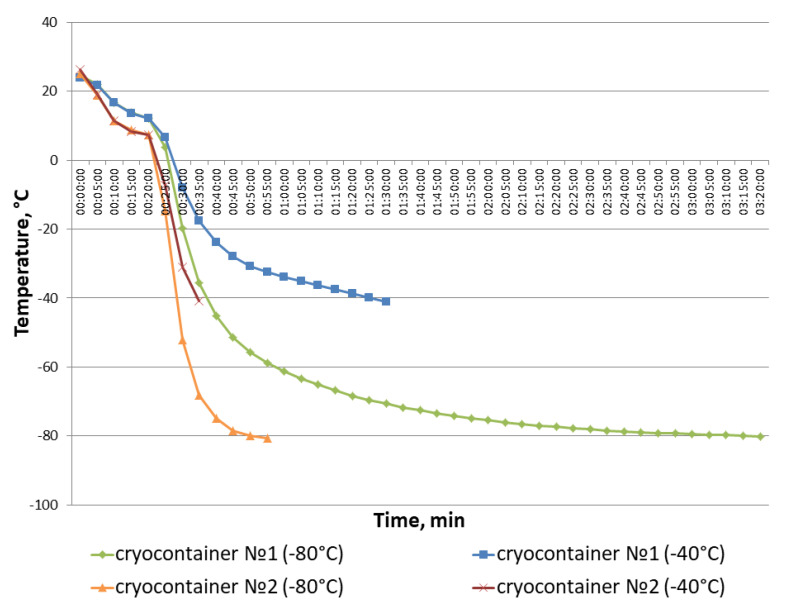
Dynamics of temperature variations in cryocontainer types No. 1 and No. 2 during the repetition of the full cycles of scaffold sample freezing until a stable cryopreservation temperature was reached.

Most often, the cryopreservation of cellular materials is based on slow freezing [21]. When running such freezing protocols, the samples are cooled at a rate of 1 °C/min [58]. A programmable freezer can provide this cooling rate, but this is expensive equipment requiring a qualified operator; moreover, liquid nitrogen is needed for its operation, requiring special conditions for storage and use. It is also worth noting that programmable freezers are not optimal for the cryopreservation of just small numbers of samples. Special-purpose cryocontainers providing a 1 °C/min freezing rate are considered to be an acceptable alternative to programmable freezers [22]. During our research, two types of thermal containers were used for cryopreservation. The thermal containers differed in their manufacturing materials and design. Both of them had to ensure uniform sample cooling at a rate of 1 °C/min. However, the differences we obtained, based on the results of scaffold sample cryopreservation, suggest that the freezing temperature regimens in cryocontainer types No. 1 and No. 2 differed. The performance of the cryocontainers used was tested. Temperature variations were recorded with the help of a thermal sensor during repeated, full cycles of scaffold sample freezing until a stable −40 °C or −80 °C cryopreservation temperature was reached (Figure 7).

The cryopreservation protocol included the samples in their cryocontainers being pre-cooled in a refrigerator (~+5 °C) for 20 min to ensure uniform penetration of the cryoprotectant into the composite structure. The containers were then moved to freezers with the relevant storage temperature. Thus, the temperature change “threshold” is visible on the graph after the 20-min point. It was found that the temperature in cryocontainer type No. 1 changed more smoothly than in cryocontainer type No. 2. So, it took an hour and a half to reach −40 °C and more than 3 h to reach −80 °C in container type No. 1. In cryocontainer type No. 2, these temperatures were reached in just 35 and 55 min, respectively. It is also worth paying attention to the smoother temperature decrease observed after the temperature change “threshold” when samples in cryocontainer type No. 1 were placed into the freezer, as represented by points 3–4 on the graph. Correspondingly, the temperature values for cryocontainer type No. 2 after the “threshold” are displayed on the graph as a straight line. The latter indicates a rather sharp temperature drop in cryocontainer type No. 2 during freezing. Thus, cryocontainer type No. 1 provided a smoother temperature decrease during sample freezing compared to cryocontainer type No. 2, causing better preservation of the cells in the scaffold during cryopreservation.

## 4. Conclusions

The presented research results demonstrate the possibility that the cryopreservation of complex tissue-engineered constructs can provide retention of both scaffold carrier integrity and of cellular component functional activity (viability, ability to exhibit three-dimensional growth, and proliferative and secretory activity) after the product is withdrawn from cryopreservation. However, the conducted studies show the critical importance of the choice of temperature regimens for freezing such TECs. It was shown that the freezing rate and storage temperature together determine the success of TEC cryopreservation, with other conditions being equal. As with the cryopreservation of cell culture suspensions, where it has been shown that an acclimatization stage increases their regenerative potential, an acclimatization stage is also necessary when removing TECs from cryopreservation. As a result of our studies, it was confirmed that acclimatization also allows for restoration of both the scaffold carrier structure, if it has “shrunk” during freezing, and the functional activity of the contained cells after removal from cryopreservation.

The number of studies dedicated to TEC cryopreservation is currently insignificant. In this regard, no single approach exists for assessing the quality of products exposed to cryopreservation. In addition, most authors have focused only on assessing the viability of the cells immediately after TEC withdrawal from cryopreservation. The results we obtained confirm that assessing the TEC’s cell viability immediately after cryopreservation is not sufficient to assess the usefulness of the tissue-engineered constructs. It was shown that cell death after product cryopreservation may be delayed and could be observed not in the first 24 h after defrosting, but during cultivation at a later stage. It is also important to evaluate not only the retention of the viability of the cells, but also the restoration of their functional activity that allows the TEC to fulfill its regenerative potential. We have therefore shown that to characterize TEC quality, it is advisable to evaluate the real-time proliferative and secretory activity of the product’s cellular component by culturing it for a period following the cryopreservation. It should be noted that the assessment of cell morphology during TEC cultivation after cryopreservation is also an important criterion, allowing for characterization of the restoration of the activity of the cellular component. The presented indicators determined after cryopreservation and compared to the same TEC indicators before cryopreservation allowed for evaluation of the effectiveness of the cryopreservation protocol.

The presented protocols and approaches to assess TEC quality before and after cryopreservation can therefore become a basis for developing cryopreservation protocols for various tissue-engineered products. The latter will make it possible to increase TEC availability for clinical use and for successful commercialization.

## Figures and Tables

**Table 1:** Assessment of viability and proliferative activity of cells in scaffolds before and after cryopreservation.

Cryocontainer		Without Freezing		After Freezing			
		72 h	144 h	−40 °C		−80 °C	
				24 h	96 h	24 h	96 h
No. 1	Total Number of Cell Nuclei per 1 mm^3^ Scaffold	404.16 ±11.19	643.65 ±18.33 **	526.28 ±9.45 ** ●●**►**	444.82 ±13.66 * **●●**	524.09 ±15.50 ** ●●►	722.48 ±26.16 ** ●
	Percent of Nuclei of Dead Cells per 1 mm^3^ Scaffold (%)	0.16 ±0.04	0.35 ±0.04	4.88 ±0.69	5.45 ±0.52	3.57 ±0.53	3.79 ±0.41
No. 2	Total Number of Cell Nuclei per 1 mm^3^ Scaffold	333.80 ±8.42	610.22 ±16.00 **	549.64 ±15.12 ** ●●►	400.44 ±8.95 ** ●●	511.09 ±12.34 ** ●	525.40 ±12.70 ** ●
	Percent of Nuclei of Dead Cells per 1 mm^3^ Scaffold (%)	0.11 ±0.03	0.28 ±0.04	3.18 ±0.34	12.69 ±1.54	3.26 ±0.48	10.56 ±1.13

Note: *—*p* ˂ 0.05; **—*p* ˂ 0.001, comparison with 72 h before cryopreservation; ●—*p* ˂ 0.05; ●●—*p* ˂ 0.001, comparison with 144 h before cryopreservation; ►—*p* ˂ 0.001, comparison with 96 h after cryopreservation. The comparison was carried out within the series “cryocontainer No. 1” and “cryocontainer No. 2”, Wilcoxon test.

## Data Availability

The data presented in this study are available in the article.
